# pH-Responsive Inulin–Sodium Alginate Hydrogel Beads for Sustained Oral Delivery of Bacterial Serine Protease

**DOI:** 10.3390/bioengineering13060655

**Published:** 2026-06-01

**Authors:** Shagufta Kamal, Sheeza Shoukat, Kanwal Rehman, Sumble Malik, Farwa Batool, Anam Ahsan, Muhammad Sajid Hamid Akash

**Affiliations:** 1 Department of Biochemistry, Government College University, Faisalabad 38000, Pakistan; 2 Department of Pharmacology, The Women’s University, Multan 60000, Pakistan; 3 School of Pharmacy and Biomedical Science, Adelaide University, Adelaide, SA 5000, Australia; 4 Department of Pharmaceutical Chemistry, Government College University, Faisalabad 38000, Pakistan

**Keywords:** serine protease, enzyme entrapment, targeted delivery, pH-responsive release, controlled release, drug release kinetics

## Abstract

To overcome the limitations of conventional therapies, targeted delivery of therapeutic interventions is crucial. Specifically, novel hydrogels maximize efficacy while minimizing the premature degradation of therapeutic enzymes. Therefore, the present study aimed to evaluate a developed pH-responsive inulin–sodium alginate (Na^+^ alginate) hydrogel bead system for colon-specific release of serine protease. Scanning electron microscope (SEM) images revealed structural differences between blank and encapsulated hydrogel beads. Fourier transform infrared spectroscopy (FTIR) spectra further confirmed the successful encapsulation of the bacterial serine protease in inulin-Na^+^ alginate (IN-Na^+^ Alg-SP). Thermogravimetric analysis (TGA) confirmed the thermal stability of hydrogel beads over a wide temperature range. Fabricated IN-Na^+^ Alg hydrogel beads displayed an entrapment efficiency of 54 ± 0.99% with an apparent activity of 260 U/mL. In vitro studies confirmed pH-responsive release with minimal release at pH 1.2 and sustained release at pH 7.4 over 4 h and 30 min. The ex vivo intestinal study confirms that the developed hydrogel has excellent potential as an oral colon-targeted drug delivery system for therapeutic enzymes. The release data best fit the second-order and Korsmeyer–Peppas models (R^2^ > 0.96), indicating a combination of diffusion and erosion-controlled release mechanisms. These findings suggest that inulin-Na^+^ alginate hydrogels provide a promising carrier system for colon-specific delivery of serine protease with potential applications in targeted protein digestion and therapeutic interventions.

## 1. Introduction

Oral administration of therapeutic proteins remains a major challenge due to their vulnerability to enzymatic degradation, instability issues in GIT conditions, and low permeability across cellular membranes. These challenges can markedly reduce the treatment effectiveness and absorption level. Thus, there is a need to design a carrier system that maintains protein stability and enables sustained release under biological conditions. The encapsulation technique has emerged as an efficient method to promote site-specific release in the gut and protect protein drugs from acidic conditions [1,2]. Encapsulation is a potential strategy to enhance the survival of protein-based drugs in the stomach and ensure that an adequate amount of the protein drug reaches the targeted site [3].

Controlled drug delivery systems utilizing natural biodegradable polymers are a rapidly evolving and highly significant scientific domain [4]. pH-responsive hydrogels are the focal point of research in this arena owing to their outstanding ability to respond to pH fluctuations [5]. Hydrogel beads, composite-based hydrogels, nanofibers, and micro-beads are gaining enormous interest due to their extensive biological and industrial applications [6,7]. Hydrogel-mediated carrier systems have been widely studied for oral therapeutic delivery owing to their biosafety, modifiable physicochemical properties, and high moisture content [8]. pH-responsive hydrogels particularly respond to environmental pH changes by exhibiting swelling and deswelling behavior, which promote enhanced release at neutral to slightly alkaline intestinal pH while enabling minimal release under acidic gastric conditions [9]. These properties make hydrogels highly effective for the regulated release of delicate biomolecules such as enzymes and proteins.

Beyond traditional polymer-based hydrogels, advanced pharmaceutical delivery systems like ionic liquids (ILs) and ionogels have received significant attention. Ionogels as hybrid materials were reviewed by Le Bideau et al. [10], who loaded organic and inorganic networks with ionic liquids, particularly polymer and silica-based ionogels, and highlighted their applications in catalysis, electrochemical devices, and drug release [10]. The use of ionic liquids as a potential tool in drug delivery systems was further illustrated by Shukla et al. [11], who demonstrated them as solubilizing agents for low water-soluble drugs, permeation enhancers, and active pharmaceutical ingredients (API-ILs). These properties of ionic liquids make them potential candidates for oral, topical, and transdermal routes [11]. The synergistic interactions of surfactant blends in non-polar and aqueous media were demonstrated by Bardhan et al. [12], showing enhanced efficacy as nanoreactors for applications in drug delivery through microemulsion systems [12]. Ghoshdastidar et al. [13] showed that high nucleobase-solubilizing ability was exhibited by the low-viscosity ionic liquid/water mixtures through hydrogen-bonding networks, which provides a thorough understanding of IL−biomolecular interactions related to drug formulation [13]. Overall, these studies elucidate that ionogels and ILs possess enhanced permeability, versatile formulation strategies, and tunable physicochemical properties; however, their clinical translation is limited due to scalability challenges, regulatory ambiguities, and toxicity concerns.

Although several natural polymers, including inulin, chitosan, sodium alginate, agar, starch, guar gum, and xanthan gum, are commonly used to prepare hydrogels [6], the quest for novel materials is still in its early stages. Among these polymers, sodium alginate (Na^+^ Alg), a natural anionic polysaccharide, is frequently used as a carrier due to its biodegradability [14], good biocompatibility [15], mucoadhesive properties [16], non-toxicity [17], mild gelation conditions, and resistance to denaturation at low pH. Moreover, its ability to generate robust hydrogel matrices and its pH-sensitive behavior with divalent cations make it well suited for oral delivery of proteins, where preservation of biomolecules in the acidic gastric medium is crucial. To reduce hydrogel porosity and increase the efficacy of protein drugs, an additional polysaccharide is introduced into the system to bind with Na^+^ Alg [18]. Inulin, “the fructose polymer”, when incorporated into Na^+^ Alg as the core matrix, improves protein stability, enhances hydrogel performance throughout the GIT [19] and potentially serves as a prebiotic [20]. Its β-(2 → 1) glycosidic linkage provides structural flexibility and chemical stability and resists enzymatic degradation until it reaches the colon [21]. Its outstanding targetability, non-toxicity, pH-responsive behavior, structural versatility, and adaptive degradation kinetics make it an exceptional carrier for pH-responsive and intestinal drug delivery applications in biomedicine and healthcare [20,21]. Herein, bacterial SP was encapsulated in IN-Na^+^ Alg hydrogel without any cross-linker, making this a cost-effective approach, and its pH responsiveness was investigated. In addition, cytotoxicity and thrombolytic potential were assessed.

## 2. Materials and Methods

### 2.1. Chemicals and Materials

Nutrient broth, ammonium sulphate, nutrient agar, casein, tyrosine, Folin Ciocalteu (2N), Bradford reagent, inulin, Na^+^ alginate, calcium chloride (CaCl_2_), hydrochloric acid (HCl), monobasic potassium phosphate (KH_2_PO_4_), dipotassium phosphate (K_2_HPO_4_), sodium carbonate (Na_2_CO_3_), sodium hydroxide (NaOH), ethanol (99%), and all other chemicals used for encapsulation were of analytical grade and were purchased from Sigma-Aldrich (St. Louis, MO, USA), unless otherwise stated.

### 2.2. Serine Protease

Serine protease previously produced from *Bacillus subtilis* MS1, having 260 U/mL [22] was used for targeted delivery. One catalytic unit (U) of serine protease corresponded to the enzyme concentration required to generate 1 µmol of tyrosine per minute under the assay protocol. The Bradford method was employed to quantify protein concentration with BSA as a standard [23].

### 2.3. Preparation of Inulin−Na^+^ Alginate Hydrogel Beads

**Figure 1:**
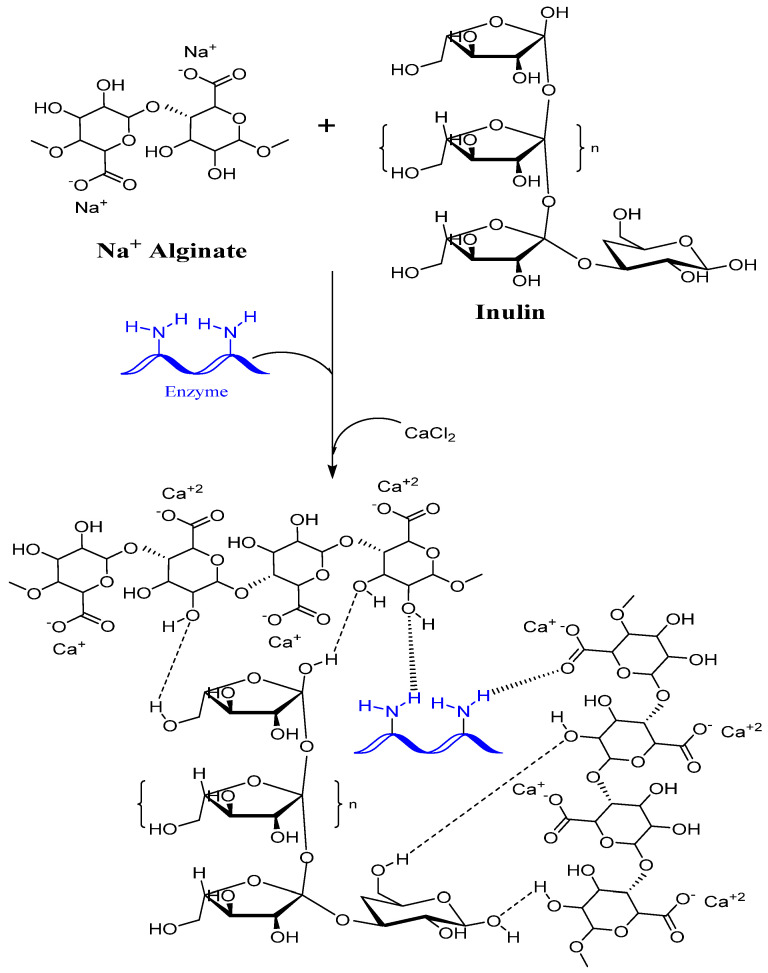
Proposed scheme of serine protease encapsulation in inulin (IN)-coated Na^+^ Alg beads.

**Figure 2:**
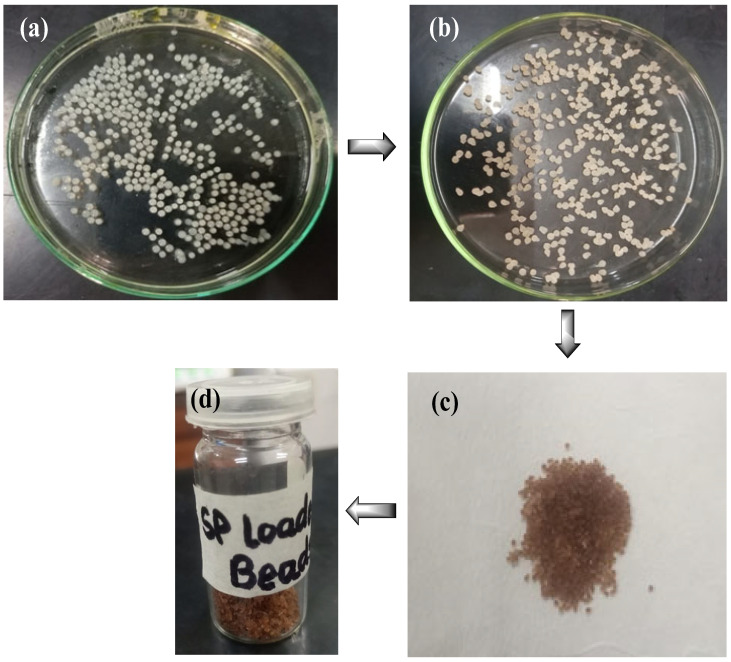
Synthesis of serine protease-encapsulated inulin sodium alginate beads. (**a**) Hardening of beads; (**b**) washing and removing excess moisture; (**c**) drying of beads; (**d**) storage of encapsulated dried beads in a glass vial.

Hydrogel beads were prepared by the ionotropic/extrusion gelling method [24]. To produce rigid beads, Na^+^ Alg (6% *w*/*v*) and inulin (3%, 6%, and 12% *w*/*v*) were dissolved in distilled water under continuous stirring until completely homogeneous. Purified serine protease was mixed into the polymer solution. The mixture was then extruded dropwise using a syringe into 40 mL of 0.2 M CaCl_2_ solution under gentle stirring (Figure 1) [25]. The beads formed immediately were allowed to cure for 30 min, then washed with distilled water and air-dried (Figure 2).

#### 2.3.1. Characterization of Serine Protease-Encapsulated IN-Na^+^ Alg Hydrogel Beads

Encapsulation efficiency (EE%) was calculated as the percentage of unentrapped enzyme in the CaCl_2_ solution during washing, determined by enzyme assay. The EE (%) was calculated according to the relation given in Equation (1):

(1) $$EE\;\left(\%\right)=\frac{Control-Supernatant\;}{Control}\;\times 100$$

Control is the total enzyme loaded into the beads, and the free enzyme leaches from them.

#### 2.3.2. Swelling Behavior Analysis

The swelling behavior of IN-Na^+^ Alg hydrogel beads was evaluated in buffer solutions of varying pH (1.2, 3.5, and 7.4) to mimic gastrointestinal conditions. Pre-weighted dry beads (~25 mg) were submerged in 20 mL of each buffer solution at 37 °C and gently shaken at 50 rpm [26]. At predetermined time intervals (0.5, 1, 1.5, …, and 4 h), beads were removed, and surface moisture was removed and promptly weighed using an analytical balance. Then, the swelling ratio [27] was measured using Equation (2):

(2) $$SR\;\left(\%\right)=\;\frac{Wt\;-Wo}{Wo}\;\times 100$$

In the equation, W_t_ is the hydrogel beads after swelling at time “t”, while the dry weight of the beads is W_o_. The analysis was conducted in triplicate, and mean values ± standard deviations were reported.

#### 2.3.3. In Vitro Release Study

The in vitro release of serine protease was investigated by incubating hydrogel beads (25 mg) in 20 mL of buffers of various pH (1.2, 3.5, 4.5, and 7.4) at 37 °C with constant agitation (50 rpm). At predetermined intervals, the release medium (2 mL) was withdrawn and replaced with a fresh buffer. The amount of enzyme released was determined spectrophotometrically at 280 nm and expressed as cumulative (%) released over time. The in vitro SP release was carried out by placing the IN-Na^+^ Alg-SP beads (25 mg) in buffers of different pH values (1.2, 3.5, 4.5, and 7.4).

#### 2.3.4. Ex Vivo Study

An ex vivo study was performed using the modified method [28,29]. In this procedure, a freshly isolated chick intestine was obtained within 1 h post-slaughter. The intestine was cleaned, washed with chilled PBS (pH 7.4), and then divided into 5–7 cm segments. Each segment was tied at one end, and hydrogel beads (~25 mg) were suspended in 1–2 mL PBS, which was then sealed at the other end. The sacs were incubated in 50 mL PBS (pH 7.4) at 37 °C under continuous shaking (50–100 rpm). The external medium (2 mL) was withdrawn and replaced with fresh buffer at predetermined time intervals. Release of enzymes was measured spectrophotometrically at 280 nm.

### 2.4. Enzyme Release Kinetics

To elucidate the release behavior of serine protease [30] from the IN-Na^+^ Alg hydrogel system, in vitro release data were fitted to five commonly used kinetic models, i.e., the zero-order model (Equation (3)), first-order model (Equation (4)), second-order model (Equation (5)), Higuchi (Equation (6)), and Korsmeyer−Peppas model (Equation (7)) [31]. The best model describing the release mechanism was determined depending on the correlation coefficient (R^2^), with a higher R^2^ value indicating a better fit.

The mathematical models used are as follows:

(3) $$M_{t}\;=\;M_{0}\;+\;K_{0}t$$

where *Mt* is the cumulative drug release amount at time interval t, *M*_0_ is the encapsulated drug, and *k*_0_ is the zero-order kinetic rate constant.

(4) $$ln\left(M_{0}-M_{t}\right)=ln\left(M_{0}\right)-K_{1}t$$

where *k*_1_ is the rate constant of the first-order model.

(5) $$\frac{1}{\left(M_{0}-M_{t}\right)}=\frac{1}{M_{0}\;}-K_{2}t$$

where *k*_2_ is the second-order rate constant.

(6) $$M_{t}=K_{H}t^{1/2}$$

where *k_H_* stands for “the Higuchi rate constant”.

(7) $$log\;\left(\frac{M_{t}}{M_{f}}\right)=log\;K_{kp}+n\;logt$$

where *M_f_* is the concentration of the therapeutic agent released at time *t*, *n* is the diffusion exponent, which determines the release system, and *k_K-P_* is the Korsmeyer−Peppas kinetic constant.

### 2.5. Determination of the Biological Potential of Encapsulated Serine Protease

Biological evaluation is crucial for determining the therapeutic efficacy and safety of encapsulated systems. The IN-Na^+^ Alg-SP beads were subjected to thrombolytic, antibacterial, and cytotoxicity assays to explore their biomedical potential.

#### 2.5.1. Thrombolytic Activity

The thrombolytic activity was assessed using a modified method described by [32]. Blood (1 mL) was immediately distributed in pre-weighed sterile tubes and incubated at 37 °C for 45 min until clot formation. The serum was separated without disruption of the clot. The weight of the clot was determined in each tube as:

Weight of the clot=Weight of tube containing clot−Weight of empty tube

Resuspended clots in the sample (1 mL) were incubated at 37 °C for 3 h. Streptokinase and PBS were used as positive and negative controls, respectively. The clots were re-weighed after fluid release to evaluate the variation in clot weight. The lysis activity was calculated as:

(8) $$Lysis\;activity\;(\%)=\frac{Initial\;weight\;of\;clot-Final\;weight\;of\;clot}{Initial\;weight\;of\;clot}\;\times 100$$

#### 2.5.2. Antibacterial Activity

The antimicrobial activity of IN-Na^+^ Alg beads and IN-Na^+^ Alg-SP beads was determined using the zone inhibition method against *Bacillus subtilis* (Gram-positive) and *Escherichia coli* (Gram-negative) [33]. Freshly prepared inocula of both *B. subtilis* and *E. coli* were swabbed on agar plates. The supernatant (40 µL) of the soaked beads was loaded into a well. A cefixime solution (1%: 1 mg in 1 mL) was employed as a positive standard. Following 24 h of incubation at 37 °C, inhibition zones were analyzed from three dimensions.

#### 2.5.3. Determination of Cytotoxicity

A cytotoxicity test was performed using the approach described by the authors [34]. Briefly, blood (6 mL) was diluted with PBS (pH 7.4; 10 mL). After complete dissolution, the solutions were centrifuged at 1700× *g* for 10 min at 4 °C. The aspiration technique was used to remove the supernatant, and pure RBC pellets were collected after washing thrice with PBS. The erythrocytes were diluted to acquire a 3% RBC suspension. The sample (100 µL) was added to the RBC solution (900 µL) in a separate Eppendorf tube and incubated for 30 min at 37 °C. Optical density [24] was measured at 575 nm using a double-beam UV-visible spectrophotometer (TU-1902 plus; Beijing Purkinje General Instrument Co., Ltd., Beijing, China) after centrifugation at 3000× *g* for 10 min. Hemolysis (%) was determined by using the following formula:

$$Percentage\;lysis\;of\;RBCs=\;\frac{\;Sample\;OD-Negative\;control\;OD}{Positve\;control\;OD}\;\times 100$$

### 2.6. Characterization

UV-Vis spectra of the serine protease were recorded at 200–800 nm using a UV-visible spectrophotometer (TU-1901plus, Beijing, China). To determine the product’s functional groups and individual samples, FTIR (Fourier transform infrared spectroscopy) was performed using a Perkin-Elmer FTIR (PerkinElmer Inc., Shelton, CT, USA) in the range of 4000–500 cm^−1^. The size of dehydrated hydrogel beads was determined by using an electronic Vernier caliper, and the average diameter was computed from 20 randomly picked beads. Morphological analysis of the hydrogel at different pH values was investigated by scanning electron microscopy (SEM; CUBE-1000; Emcraft, Republic of Korea). Thermal analysis was carried out using a TA Instruments Q600 thermogravimetry-differential scanning calorimetry (TGA-DSC) thermal analyzer (TA Instruments, New Castle, DE, USA), which was preliminarily calibrated with an indium standard. Dry samples (8.17 ± 0.1 mg) with a nitrogen flow (100 mL/min) were heated in an alumina pan up to 1000 °C (temperature) at 20 °C min^−1^.

### 2.7. Docking Analysis

The potential mode of interactions between a serine protease receptor (PDB ID: 1HXE) and sodium alginate/inulin was explored by performing docking analysis. Discovery studio (v21.1.0.20298) was used for the preparation of the protein and its minimization by eliminating heteroatoms, non-essential chains, and water molecules. While Avogadro was used for building ligand structures, their minimization was carried out in PyRx, followed by export in PDBQT format using Open Babel (PyRx; virtual screening tool software). In PyRx, AutoDock Vina 4 was used for performing docking with a search space covering the active site area, with its exhaustiveness set to 10. The docking scores were recorded in Kcal/mol, and the results were taken as predictive, without providing any experimental confirmation, as reported previously [35].

### 2.8. Statistical Analysis

All experiments and characterization analysis were conducted in triplicate, and results are expressed as mean ± S.D. Data were statistically analyzed using Origin 6.0 software and Microsoft Excel (Origin Lab Corporation, Northampton, MA, USA).

## 3. Results

### 3.1. Hydrogel Development

The results of different formulations of IN-Na^+^ Alg hydrogel beads are exhibited in Table 1. At a lower inulin concentration (3%), fragile and soft beads resulted in an unsuccessful trial. However, increasing the inulin concentration up to 6% resulted in rigid spherical beads. Hard beads were formed using 12% inulin. Consequently, a 1:1 formulation was optimized and used for further encapsulation studies. Similar data have been reported in an alginate−inulin composite system in which inulin concentration (up to 10%) gives denser structures by hydrogen bonding, smooth surfaces, resistance to GIT conditions, and improved mechanical stability [36]. Wang et al. (2024) [7] reported that incorporating inulin into alginate matrices increases stability, bead diameter, and structural compactness, making it suitable for applications of GIT delivery systems; however, high inulin levels may soften the beads by hindering cross-linking and increasing feed viscosity. Based on bead morphology and mechanical integrity, a 1:1 formulation (6% inulin/6% alginate) was selected as the most suitable optimized composition for further encapsulation studies because it provided adequate rigidity without excessive hardness, which may impede kinetics.

#### 3.1.1. Enzyme Encapsulation

**Figure 3:**
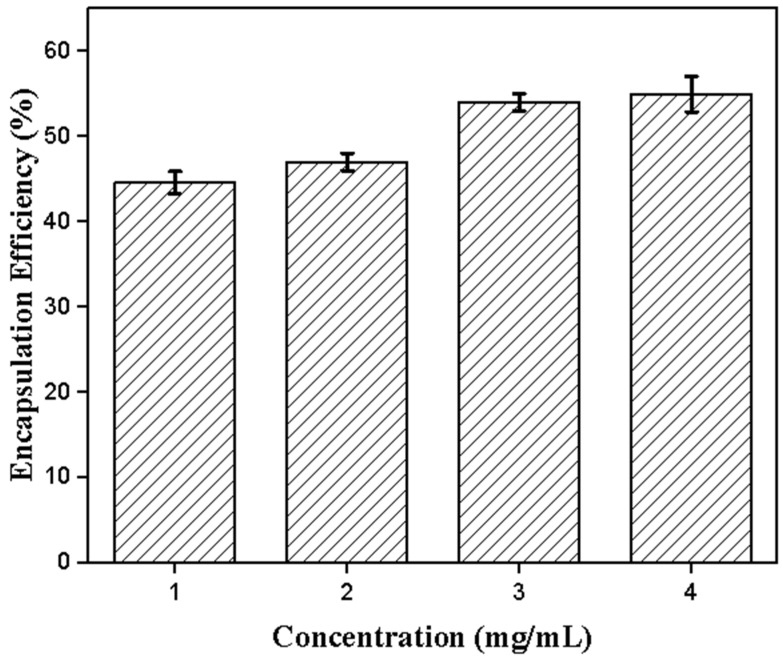
IN-Na^+^ Alg hydrogel bead-loading efficiency of different concentrations of serine protease.

The encapsulation efficiency of hydrogel beads demonstrated a significant improvement in enzyme retention, 44 ± 1.31%, 47 ± 1.023%, and 54 ± 1%, respectively, as the concentration of beads (1 mg/mL to 3 mg/mL) rose. However, no statistically significant relation between enzyme retention (%) and concentration was observed. When the concentration was 3 mg/mL, the retention (%) was 54 ± 1%, while at 4 mg/mL, 55 ± 2.08% was observed, as shown in Figure 3. No significant improvement may be due to limited entrapment sites within the Ca^+2^-cross-linked alginate−inulin network, also indicating saturation of the polymeric network [37]. Encapsulated protease in alginate-chitosan beads also showed concentration-dependent behavior, with an optimal point beyond which bead formation inefficiencies lead to low retention [37,38]. Thus, 3 mg/mL was selected as the optimized enzyme loading concentration for the following studies.

#### 3.1.2. Swelling Index

**Figure 4:**
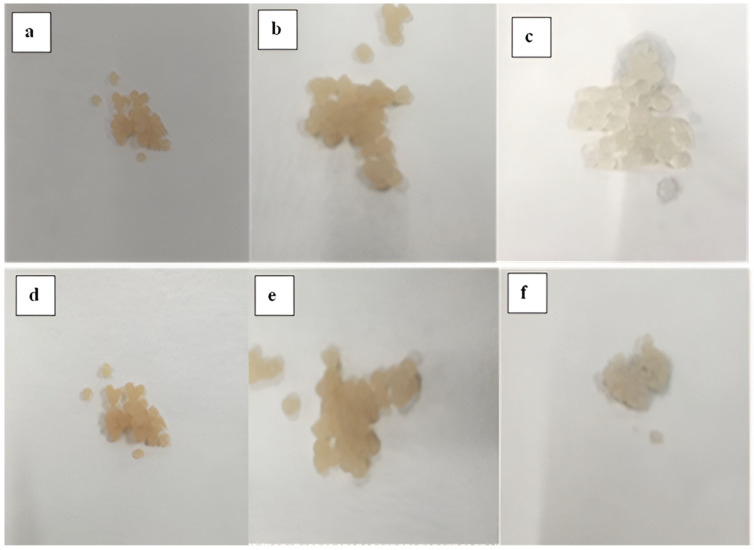
Swelling indices of IN-Na^+^ Alg-SP hydrogel beads at pH values of 1.2 (**a**), 3.5 (**b**), and 7.4 (**c**) after 30 min and at pH values of 1.2 (**d**), 3.5 (**e**), and 7.4 (**f**) after 120 min.

**Figure 5:**
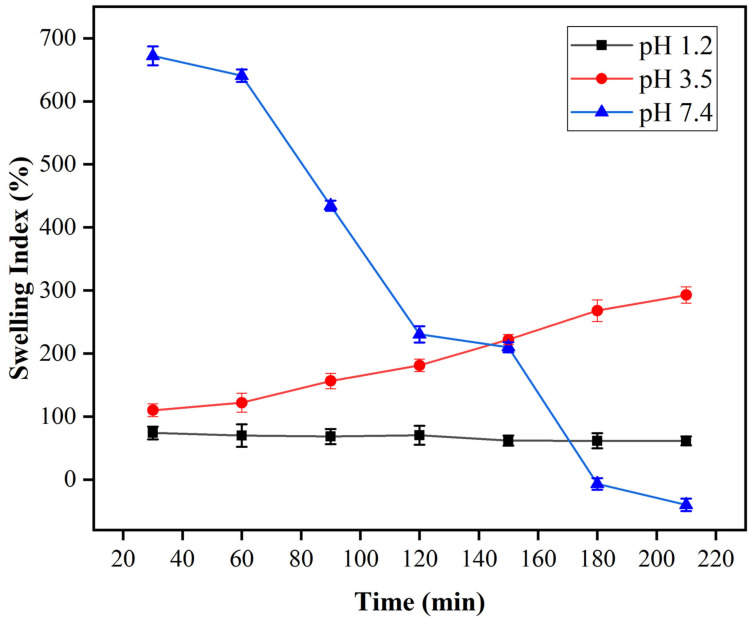
Swelling indices of IN-Na^+^ Alg-SP hydrogel beads at different pH.

The stability and swelling dynamics of the IN-Na^+^ Alg-SP hydrogel beads, upon exposure to various buffer treatments, illustrate that swelling was subdued under pH 1.2 (acidic medium), in contrast to pH 3.5 and 7.4 at different time intervals, as presented in Figure 4. However, in PBS (pH 7.4), the IN-Na^+^ Alg-SP hydrogel exhibited extensive swelling, attributed to the loss of Ca^2+^ ions and subsequent hydrogel degradation (Figure 5). The massive swelling in PBS (pH 7.4) was attained by partial deterioration of beads (Figure 5). Swelling reduction under acidic conditions (pH 1.2) resulted due to protonation of alginate carboxyl groups (–COO^−^ → –COOH), leading to limited water uptake and shrinkage of the polymer network [9]. The pH-responsive profile is consistent with recent alginate and inulin-modified hydrogels for colon/intestinal delivery, lowering gastric swelling while increasing release at higher pH [39]. IN-Na^+^ Alg-SP hydrogels are suitable for intestinal enzyme release and gastric protection [9].

The color variations in Figure 4c vs. Figure 4a/b) were not attributed to drug loading since the samples were of the same batch. They were rather caused by swelling of hydrogels at different pH during an exposure of 30 min. At pH 1.2, the brown color was preserved by limited swelling; at pH 3.5, partial ionization caused moderate swelling as the pigments were dispersed to give a lighter brown color; at pH 7.4, extensive swelling diluted the pigments to give transparency. Therefore, the changes observed were due to pH-responsive hydration behavior, not due to drug loading changes.

#### 3.1.3. pH-Responsive Release

**Figure 6:**
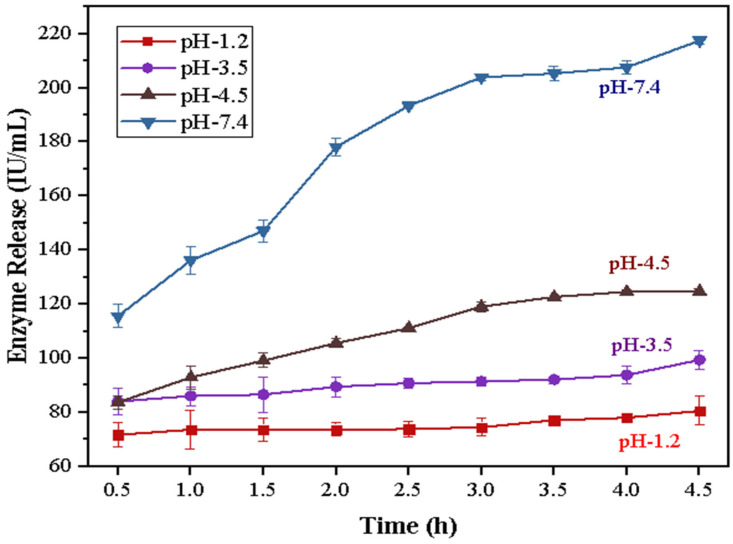
In vitro SP release at varying pH levels after different time intervals (0.5 h to 4.5 h).

A pH-dependent SP release pattern was observed by the IN-Na^+^ Alg-SP hydrogel at four different pH values (pH: 1.2, 3.5, 4.5, and 7.4). In vitro SP release results showed that only 5% of the SP was liberated at pH 1.2 throughout the incubation period. At pH 3.5 and 4.5, relatively small amounts of SP were released, whereas a substantial amount was released at pH 7.4, reaching a maximum within 4 h (Figure 6). However, negligible release at acidic pH was due to limited diffusion into the reduced hydrogel network, aligning with previous data showing an alginate-based delivery system suppressed drug/protein release at acidic pH. At pH 1.2, alginate hydrogels maintained integrity by limiting payload release, whereas at pH 7.4 enhanced and rapid release is consistent with deprotonation-mediated matrix expansion [9]. At pH 7.4, higher release can be due to enhanced hydrogel swelling and partial weakening of calcium ions’ ionic cross-links, promoting diffusion of entrapped proteins, a widely documented mechanism in pH-responsive hydrogel carriers [40]. In acidic environments (pH: 1.2, 3.5, and 4.5), the hydrogel retained the enzyme and minimal enzyme leakage was demonstrated throughout the incubation period. The hydrogel was thus rendered as acid pH sensitive. However, at pH 7.4, the enzyme release reached a maximum at 4.5 h, and no significant variation was recorded after 3 h, which showed that the plateau was reached and the release rate was constant thereafter. Moreover, the hydrogel beads were disintegrated at pH 7.4, which caused the maximum release of the enzyme during this period; hence, 4.5 h was taken to be adequate to define the release pattern of the system [37]. It can be concluded that the IN-Na^+^ Alg hydrogel protects SP in gastric conditions while allowing controlled release at intestinal pH.

#### 3.1.4. Ex Vivo Study

**Figure 7:**
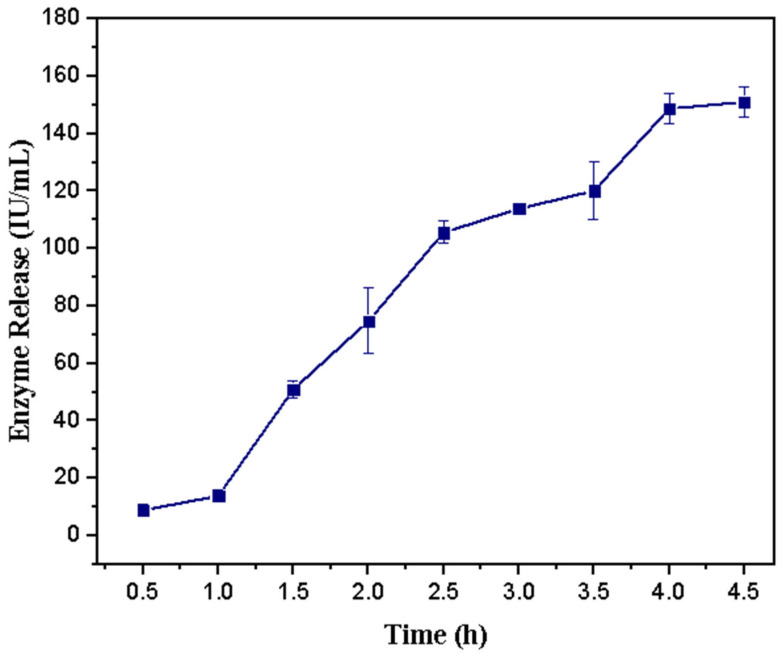
Ex vivo SP release at different time intervals (0.4 h to 4.5 h).

The ex vivo intestinal release profile of SP from IN–Na^+^ Alg hydrogel beads exhibited a gradual and sustained increase in enzyme release over time under physiological intestinal conditions (Figure 7). Initially, minimal SP release occurred during the first hour of incubation, whereas substantial release was observed after 1.5–2 h, eventually reaching a maximum at 4.5 h. This release behavior demonstrates that the developed hydrogel system enables controlled sustained diffusion of the enzyme in a biologically relevant environment of intestine, avoiding burst release. The controlled release of enzyme was attributed to gradual swelling of the IN-Na^+^ Alg matrix at intestinal pH, which allowed the enzyme to diffuse through the hydrogel network.

The observed SP release pattern confirms in vitro pH-responsiveness, where SP retention is high in an acidic environment and pronounced release of the enzyme at neutral conditions (pH 7.4). However, under ex vivo conditions, the release pattern was relatively slow, which may be due to intestinal mucus, diffusion barriers associated with tissues, and interactions between the released enzyme and components of the intestinal membrane. This kind of behavior is beneficial for oral protein delivery systems as it results in more prolonged release in the intestine, which increases the time of action of proteins and can increase local bioavailability.

A significant increase in SP release after 2 h further demonstrates that the hydrogel matrix did not disintegrate significantly during the initial incubation period but continued to swell. The swelling of the IN-Na^+^ Alg hydrogel at neutral intestinal pH might be due to deprotonation of carboxylic groups, which results in electrostatic repulsion between polymer chains, increased hydration, and matrix expansion, which in turn leads to controlled SP release. The similar sustained intestinal release has been observed in alginate-based oral delivery systems for proteins, probiotics, and nanoformulations that maintain their integrity in the stomach and release the payload primarily in the intestine [41,42].

Furthermore, the ex vivo results further validate the biological relevance of the IN-Na^+^ Alg hydrogel and its applicability for intestinal-targeted oral delivery of SP. Recent studies also confirmed the significance of inulin and Na^+^ alginate hydrogels while explaining their ability of delayed drug release and retention in the intestine under ex vivo and in vivo gastrointestinal conditions, which might be associated with the mucoadhesive and pH-responsive properties of the hydrogel [43,44].

The results of the present study demonstrate the potential of the IN-Na^+^ Alg hydrogel beads to protect the SP enzyme through gastrointestinal transit and to release them in a controlled manner within the intestinal area. In conclusion, the ex vivo intestinal study confirms the pH-dependent drug release mechanism suggested from the in vitro study and further establishes biological significance of the developed hydrogel system, highlighting its remarkable potential as an oral intestinal-targeted drug delivery system for therapeutic enzymes.

#### 3.1.5. Entrapment Efficiency of Serine Protease on Inulin−Na^+^ Alginate Beads

The results in Table 2 reveal that 54% of the initial 3 mg of SP was efficiently entrapped within IN-Na^+^ Alg beads. The apparent activity of the entrapped enzyme was 260 U, with a specific activity of 3.12 U/mg and a specific activity of the entrapped enzyme at 3.22 U. Notably, the entrapped enzyme displayed a relative activity of 103%, indicating minimal loss of enzymatic function during encapsulation (Table 2). These results showed that IN-Na^+^ Alg bead formation preserves catalytic function and retains a substantial portion of the loaded enzyme, rendering it a viable carrier for those applications that require enzyme stability under changing pH conditions. Similar biopolymer immobilization studies have reported ≥100% relative activity, attributed to restricted enzyme movement within the matrix, which minimizes autolysis and conformational instability [45].

### 3.2. Serine Protease Release Kinetic Models

**Figure 8:**
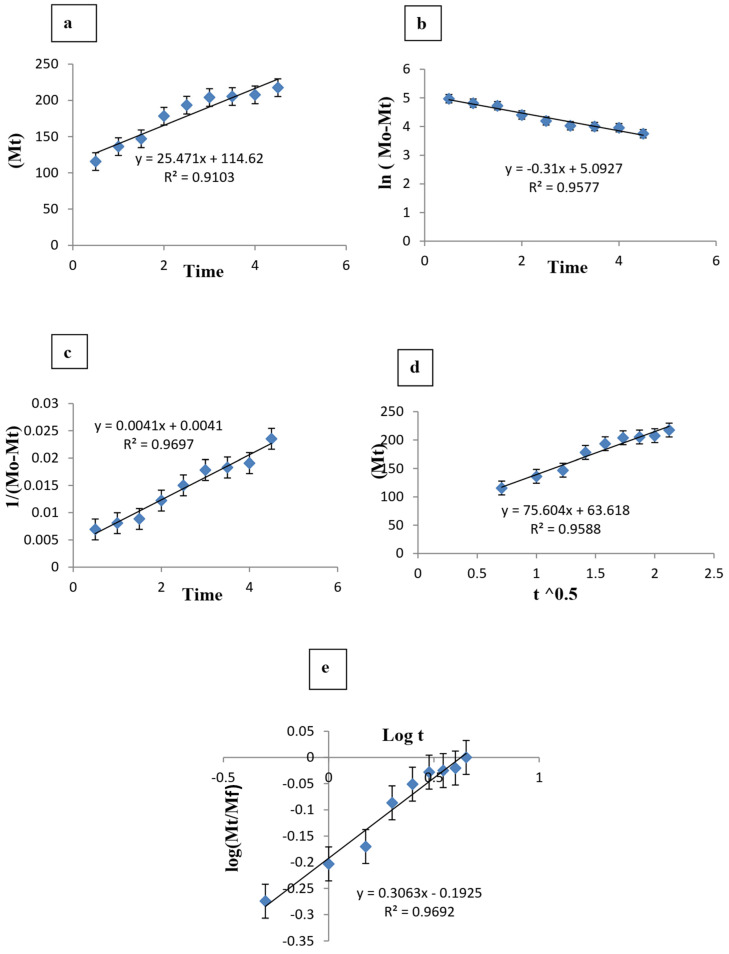
Comparison of SP release via five different models: (**a**) zero-order; (**b**) 1st-order; (**c**) 2nd-order; (**d**) Higuchi; (**e**) Korsmeyer−Peppas.

The kinetic data for SP release from IN-Na^+^ Alg hydrogel beads were fitted to five different kinetic models, and the R^2^ values of all models are presented in Table 3. Second-order and Korsmeyer−Peppas models were the best-fitting models for the SP release based on higher regression values. The lowest R^2^ value was observed for the zero-order model (Figure 8), indicating a poor fit. It suggests that serine protease release was not consistent over time but was instead concentration-dependent. The best-fit second-order model showed that residual enzyme concentration and matrix structure within the hydrogel influence the release concentration. The same data from zero-order kinetics have been observed for alginate-based pH-responsive hydrogels, where the release is primarily regulated by matrix relaxation and diffusion, instead of a constant rate [31]. The diffusion-controlled release mechanism was further supported by the Korsmeyer–Peppas model. To differentiate between erosion/polymer relaxation and Fickian diffusion, the Peppas model is widely applied to polymeric systems [46]. In alginate composite hydrogels, Peppas kinetics commonly follow release behavior due to the gradual weakening of ionic cross-links and swelling-induced diffusion under intestinal pH conditions [9]. The negative values found in the Korsmeyer–Peppas model were due to the logarithmic transformation. Since the fractional release (Mt/Mf) is less than unity during most of the release period, the logarithm of this value is negative. This is a natural mathematical consequence of the model and does not show an abnormality in the release behavior. These trends are typical of controlled release systems especially in the first stages where there is low release of the enzyme [47]. This study confirms that serine protease release from IN-Na^+^ Alg hydrogel beads is regulated by diffusion as a swollen polymer matrix with input from structural relaxation at higher pH.

All the release and kinetic modeling experiments were conducted at pH 7.4, as this was the desired physiological state for which the system was developed. Thus, release studies and kinetic modeling were carried out solely at pH 7.4. The other pH conditions (pH: 1.2, 3.5, and 4.5) were not considered in the kinetic modeling, as they were only used in preliminary stability and comparative release evaluations.

### 3.3. Physical Characterization

#### 3.3.1. FTIR Analysis

**Figure 9:**
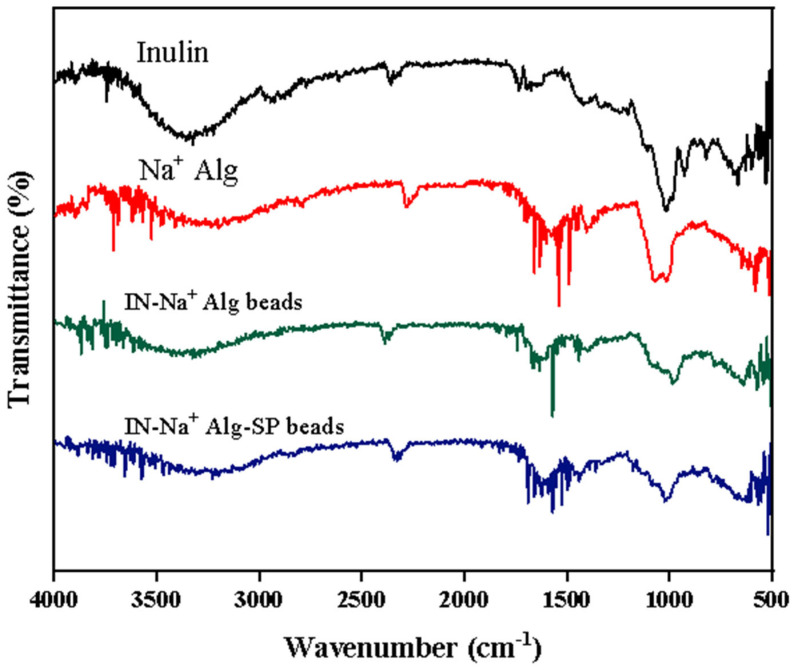
FTIR spectra of IN-Na^+^ Alg-SP hydrogel beads.

FTIR spectra of inulin, Na^+^ Alg, blank beads, and loaded beads were recorded on the FTIR spectrometer in the range of 500–4500 cm^−1^ using the transmission mode. FTIR spectra of Na^+^ Alg displayed the following characteristic absorption peaks: a broad absorption band at 3253 cm^−1^ attributed to –OH stretching (Figure 9). Additionally, distinct peaks at 1660 cm^−1^ and 1630 cm^−1^ indicate CC stretching. Furthermore, absorption bands at 1543 cm^−1^, 1490 cm^−1^, and 1406 cm^−1^ correspond to –COO vibrations. Moreover, a band at 1076 cm^−1^ signifies –CO stretching vibrations, and an absorption band at 1015 cm^−1^ is associated with C-C-C vibrations. The reduction in sharpness of the –OH group at 3335 cm^−1^ and –CH stretching vibration at 2940 cm^−1^ of inulin within the polymer hydrogel might be due to inulin with Na^+^ Alg interaction. These features align with previously reported FTIR alginate profiles [15,17]. Intensity changes and peak broadening have been reported in the inulin−alginate system, having intermolecular interactions without forming more covalent bonds [21]. The FTIR spectra of the loaded beads displayed distinct peaks at 1580–1650 and 1650–1700 cm^−1^, corresponding to the enzyme’s amide bonds, specifically the N-H and C=O groups. Similar results have been observed for enzyme-loaded alginate hydrogels, while the absence of new peaks verifies the structural compatibility between the matrix and the enzyme [8].

#### 3.3.2. Bead Size Analysis

The relatively uniform formation of beads via extrusion-based ionotropic gelation was indicated by the average bead size of the synthesized IN-Na^+^ Alg hydrogel beads observed as 0.92 ± 0.08 mm (*n* = 20). The limited size variation and spherical structure may be associated with regulated ionic cross-linking in the CaCl_2_ solution and optimal viscosity of the polymer solution. The encapsulation efficiency, release kinetics, and swelling behavior of hydrogel-based delivery systems were influenced by the bead size, considered an essential parameter.

The previously reported alginate-based hydrogel systems prepared using extrusion techniques was comparable to the obtained bead size. Bennacef et al. [48] reported that the diameter of beads generally ranges within millimeter-scale dimensions and the size of alginate beads is greatly affected by alginate properties, concentration of CaCl_2_, and conditions of extrusion [48]. Similarly, extrusion-based alginate pectin hydrogel particles were produced by Rysenaer et al. [49] that range in size from 1.27 to 1.59 mm, influenced by nozzle dimensions and concentration of polymer [49]. The comparatively homogeneous distribution of beads reported in the present work may support sustained release behavior and reproducible pH-sensitive swelling.

#### 3.3.3. Scanning Electron Microscopy (SEM)

**Figure 10:**
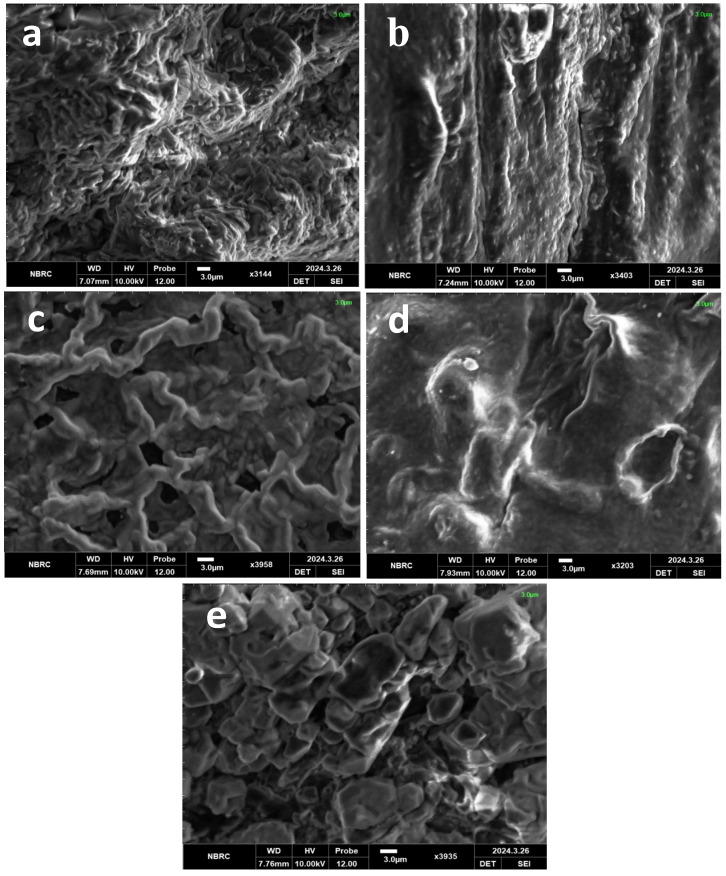
SEM images of blank beads (**a**), IN-Na^+^ Alg-SP hydrogel beads (**b**), and IN-Na^+^ Alg-SP hydrogel beads (**c**) at pH 1.2 (**d**), pH 3.5 (**e**), and pH 7.4.

The SEM analysis of blank beads and IN-Na^+^ Alg-SP beads before and after exposure to buffers of different pH is presented in Figure 10. A relatively spherical morphology with folded and rough surfaces was exhibited by the native hydrogel beads. The wrinkles, cracks, and surface roughness of IN-Na^+^ Alg were due to a partial gel network that disintegrated during dehydration procedure. The average pore diameter of the gel surface was found to be within the range of 5 to 20 µm. Notably, in IN-Na^+^ Alg-SP beads, the surface roughness was reduced, possibly due to its interaction with the enzyme and even distribution that enhanced polymer arrangement before pH exposure and reduced surface porosity levels. At pH 1.2, small pores in the hydrogel appeared due to deprotonation of the carboxylic group and the phenomenon of swelling. At pH 3.5, the hydrogel pore size increased due to buffer penetration. On the other hand, at pH 7.4, almost all the carboxylic groups of the hydrogel were deprotonated, the hydrogel structure was lost, and the beads exhibited a loss in activity. These were ascribed to polymer phase separation and structural rearrangements that occurred during drying and the sample setup for SEM imaging [37]. The smooth surface of IN-Na^+^ Alg-SP beads was mostly due to enzyme interaction and uniform distribution within the hydrogel network, which could reduce surface porosity and polymer packing before pH treatment. Since protonation of the carboxylic group decreased electrostatic repulsion, limiting water uptake and resulting in minor pore creation, the appearance of these tiny pores at pH 1.2 was suggestive of limited swelling. Higher buffer penetration enabled large pore sizes, improved carboxylate group deprotonation, and induced osmotic swelling as beads were exposed to pH 3.5. The pH-responsive behavior is consistent with findings in alginate-based hydrogels, where repulsion between deprotonated anionic groups causes maximum swelling in neutral to alkaline conditions, resulting in bead erosion and network relaxation [50].

#### 3.3.4. Thermal Analysis

**Figure 11:**
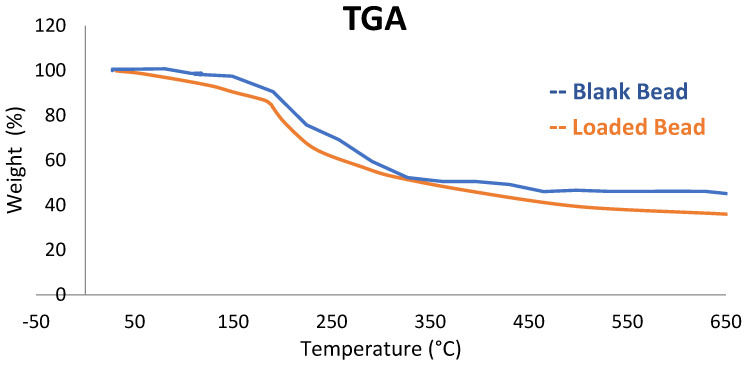
Thermogravimetric analysis of IN-Na^+^ Alg blank and SP-loaded hydrogel beads.

Thermal analysis of IN-Na^+^ Alg blank and SP beads was conducted via TGA, as shown in Figure 11. The degradation profile was slightly lower for the enzyme-loaded IN-Na^+^ Alg hydrogel, which might be due to the presence of the enzyme and slight alterations in polymer–polymer interactions within the cross-linked network, resulting in lower matrix compactness [51]. The TGA of both the hydrogels showed slight variation in thermal behavior with continuous weight loss up to 650 °C. The weight loss was categorized into two stages. In the first step, approximately 20% of the weight loss occurred at 100 °C, which might be attributed to the loss of water molecules and hydroxyl groups present in the sample. In the second step, 60% of the weight loss occurring at 150–300 °C was due to the decomposition of carbon chains of the co-polymeric material. An integrated copolymer structure collapsed at 465 °C. A maximum mass loss was observed at 600 °C. It aligns with the literature, as alginate networks in this temperature range undergo main chain breakdown and depolymerization [50]. However, 44% of the IN-Na^+^ Alg hydrogel bead and 36% of IN-Na^+^ Alg-SP hydrogel beads remained in ash residues as the weight loss became constant. The evaporation of bound and physically adsorbed water molecules and the loss of hydroxyl groups, a frequent phenomenon seen in alginate and other types of hydrogels under heat examination, are responsible for the first-stage weight loss of approximately 20% that occurred up to ~100 °C [52]. These patterns include the formation of stable char/ash and high-temperature decomposition [53]. The structural ability and distinct thermal degradation behavior characteristic of cross-linked inulin/alginate hydrogel networks are generally supported by the TGA behavior. The comparable degradation behavior of hydrogels suggests that the enzyme encapsulation did not significantly affect the hydrogel’s structural integrity.

### 3.4. Biological Activities

#### 3.4.1. Thrombolytic Activity

**Figure 12:**
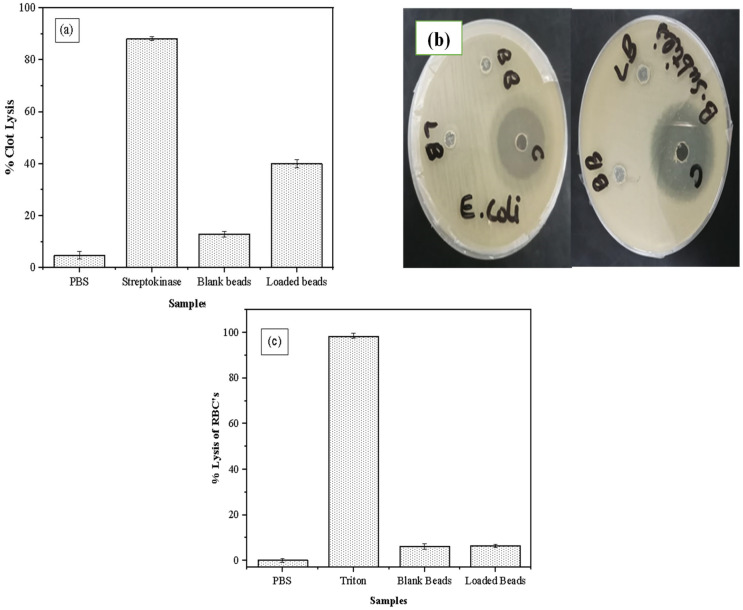
Biological activities of IN-Na^+^ Alg hydrogel beads: (**a**) thrombolytic activity; (**b**) antibacterial activity; (**c**) cytotoxicity study.

The clot lysis rate of blank beads was lower (12.90%) than that of the standard (Figure 12a). The IN-Na^+^ Alg-SP beads demonstrate relatively high thrombolytic activity, with about 40% attributed to the enzyme. However, IN-Na^+^ Alg-SP beads showed a markedly higher clot lysis (~40%), which could be due to the activity of immobilized protease causing the breakdown of the fibrin protein network within the clot. These findings correspond with the literature showing that enzyme-loaded hydrogel carriers can increase clot degradation as compared to inert control, although they have a lower potency compared to conventional thrombolytics due to diffusion limitation and controlled release. Alginate-based hydrogels serve as an effective platform for immobilizing enzymes while retaining activity, thus facilitating localized therapeutic effects in biomedical applications [54]. PBS was used as a negative control exhibiting minimal or no thrombolytic activity. Streptokinase, used as a positive control, demonstrates maximum clot lysis of 88.28% [55].

#### 3.4.2. Antibacterial Activity

The antibacterial activity of IN-Na^+^ Alg-SP hydrogel beads was evaluated through the well diffusion method, against both gram +ve and gram -ve bacteria, with cefixime as the positive control (Figure 12b). This is evident from Figure 12b, where no zones of inhibition were observed for test samples against both gram +ve and gram −ve bacteria. IN-Na^+^ Alg-SP hydrogel beads showing no antibacterial activity might be because of several factors: (1) the immobilized enzyme in the hydrogel might not diffuse properly into an agar medium from the beads; (2) biodegradable hydrogel matrices did not release active antimicrobial agents as they swelled by absorbing water, causing lack of inhibition zones; (3) enzyme protease did not show significantly bactericidal properties except if combined with nanoparticles like Ag or ZnO or antibacterial additives, while studies incorporating nanoparticles or metal ions into alginate hydrogels showed antibacterial inhibition zones in response to gram-positive and gram-negative bacteria, highlighting the role of functional additives in enhancing antibacterial effectiveness [56].

#### 3.4.3. Cytotoxicity Study

The interaction of hydrogels with RBCs was elucidated via blood biocompatibility (Figure 12c). The cytotoxicity values of the negative control (PBS), blank beads, and IN-Na^+^ Alg-SP beads were 0.02%, 4%, and 5% hemolysis, respectively, suggesting it as safe and non-toxic for in vivo applications. The cytotoxicity of the positive control (Triton-X-100) was 98.02%, confirming it as a potent hemolytic agent. Alginate-based hydrogels with enzyme-loaded variants uniformly showed <5% hemolysis, validating compatibility for biomedical applications such as wound healing and drug delivery [57].

The higher clot lysis (~40%) compared to RBC lysis (~10%) for IN-Na^+^ Alg-SP hydrogel beads was attributed to differential substrate susceptibility. The SP shows increased activity towards the porous fibrin network, allowing an efficient interaction of the enzyme and the substrate, but the lipid-rich composition and highly organized cytoskeletal structure of erythrocyte membranes inhibit the access of enzymes, thereby not promoting the lysis of erythrocytes [58]. It was this structural shielding that renders RBC membranes more resistant to the action of serine proteases as compared to fibrin networks, which were more permeable and accessible to enzymes.

### 3.5. Docking Analysis

#### 3.5.1. Inulin−Serine Protease Complex

**Figure 13:**
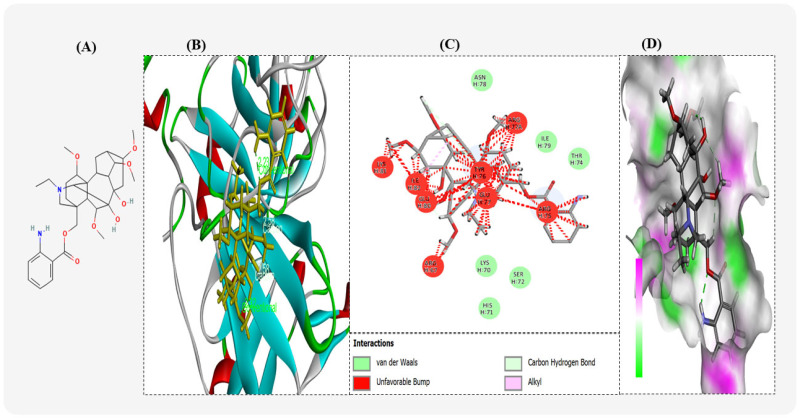
Molecular docking analysis of inulin with serine protease (PDB 1D: 1HXE): (**A**) the 2D structure of the docked inulin-associated compound (ligand); (**B**) 3D ligand-binding conformation in the active pocket of serine protease that illustrates the accommodation of the ligand in the catalytic cavity; (**C**) 2D interaction map illustrating unfavorable steric interactions, hydrogen bonding, van der Waals interactions, and hydrophobic contacts between the surrounding amino acid residues like Lys245, Gln239, Leu244, Ile240, Trp235, Arg241, Val228, and Asn246 and the ligand; (**D**) surface representation of the protein−ligand complex showing the distribution of polar and hydrophobic interaction regions and spatial occupation of the ligand in the active site pocket.

Inulin showed favorable accommodation in the catalytic binding pocket of the serine protease (PDB ID: 1HXE) as demonstrated by molecular docking, indicating structurally stable interactions between the protein and the ligand (Figure 13). A network of hydrogen-bonding interactions stabilizes the docked complex, involving residues like Ile240, Asn246, Arg241, and Gln239, which contribute to conformational stabilization and anchoring of the ligand within the active site. Moreover, ligand positioning was further reinforced through nonpolar interactions and van der Waals interactions by contacting hydrophobically with Leu244, Val228, and Trp235. The enhanced affinity of the ligand polar functional groups was suggested by observing the electrostatic stabilization via positively charged residues, specifically Arg241 and Lys245.

The ligand present in the catalytic groove was stabilized through the extensive hydrogen-bonding network, while the prolonged residence time in the binding cavity was contributed to hydrophobic interactions. The protease activity was ultimately reduced due to occupying active pocket that interferes with catalytic triad dynamics and may sterically hinder the entry of the substrate. The downstream biological effects can be contributed by such inhibitions like tissue injury, protease-mediated oxidative damage, and inflammatory amplification.

#### 3.5.2. Sodium Alginate–Serine Protease Complex

**Figure 14:**
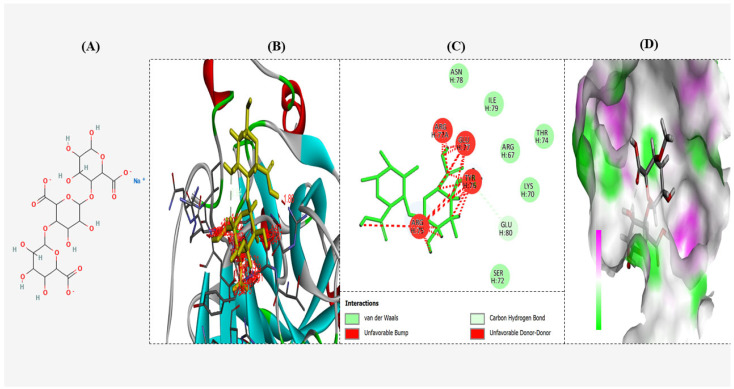
Molecular docking analysis of sodium alginate with serine protease (PDB ID: 1HXE): (**A**) Two-dimensional chemical structure of sodium alginate used for molecular docking analysis. (**B**) Three-dimensional representation of the docked sodium alginate–serine protease complex showing ligand accommodation within the active-site cavity of the enzyme. Hydrogen-bonding interactions and close-contact residues surrounding the ligand are illustrated within the catalytic pocket. (**C**) Two-dimensional interaction map depicting hydrogen bonds, electrostatic contacts, van der Waals interactions, and unfavorable steric interactions between sodium alginate and active-site residues, including Gln239, Ile240, Arg241, Asn246, Val228, Trp235, Val238, Leu244, and Lys231. (**D**) Surface visualization of the ligand–protein complex demonstrating spatial occupancy of sodium alginate within the serine protease binding cavity and distribution of hydrophilic and hydrophobic interaction regions.

The stable accommodation in the active site cavity of the serine protease (PDB ID: 1HXE) was exhibited by sodium alginate as demonstrated in molecular docking analysis, supporting favorable interaction of protein-ligand dynamics (Figure 14). Multiple hydrogen-bonding interactions were involved in stabilizing the docked complex occupying residues like Asn246, Gln239, Arg241, and Ile240, contributing to ligand anchoring in the catalytic groove. The conformational stabilization was further enhanced by additional hydrophobic interactions involving Leu244, Val228, Val238, and Trp235 via van der Waals contacts in the binding pocket. Notably, the negatively charged carboxylate groups of sodium alginate appeared to interact with the positively charged residues like Arg241 and Lys231, which suggests electrostatic complementarity that may strengthen retention of the ligand inside the active site. The sodium alginate structural compatibility in the substrate recognition area of the enzyme was supported by surface binding analysis that further indicates substantial spatial occupation of the catalytic activity.

The prolonged residence of the ligand in the binding pocket was contributed by the hydrophobic contacts, while the electrostatic interactions and extensive hydrogen-bonding network stabilize the ligand in the catalytic cleft. The substrate entry may be obstructed by sodium alginate due to its negatively charged polysaccharide structure and change the orientation of catalytic residues, thus reducing the proteolytic activity. The downstream protease-mediated biological processes can be subsequently attenuated by this inhibitory interaction, which includes extracellular matrix degradation, cellular damage associated with excessive activation of serine protease, oxidative stress amplification, and inflammatory tissue injury.

## 4. Discussion

In the present study, the hydrogel beads were optimized by adding different concentrations of inulin to the Na^+^ Alg core material. Inulin augmented the hydrogel’s viscosity, enhancing the stability and effectiveness in low-pH environments. Its distinct oligo- or polysaccharide structure, lacking sugar rings in its backbone, enables increased molecular flexibility. This property results in stronger interactions with proteins at acidic pH levels, effectively preventing protein release. Our results agree with the authors [3,37], who independently loaded 3 mg/mL protein into the IN-Na^+^ Alg hydrogel. The SP-loaded hydrogel, when exposed to buffers of varying pH (e.g., 1.2, 3.5, 4.5, and 7.4), demonstrated pH-responsive behavior with minimal SP release at pH 1.2, due to contraction of protonated carboxyl groups. As a result, water does not penetrate the hydrogel structure, thereby diminishing the swelling rate. This unique behavior makes the hydrogel suitable for protecting drugs in the stomach’s acidic conditions. As the pH increased, the encapsulated SP was completely released within 4 h and 30 min. This release of SP can be attributed to the deprotonation of carboxyl groups of Na^+^ Alg, leading to hydrogel ionization. This potential suggests its utility in colon-specific drug delivery [3,26,59].

The achieved medium encapsulation efficiency (~54%) may be linked to restricted binding sites in the polymer matrix, as well as enzyme diffusion into the external CaCl_2_ phase. Furthermore, the entrapment efficiency can be influenced by the fixed concentration of CaCl_2_ (0.2 M), which may not ensure sufficient network cross-linking. The encapsulation performance can be further improved by subsequent optimization of gelling conditions. SP kinetic profiles were suited to five distinct kinetic models, i.e., zero-, first-, second-order, Korsmeyer-Peppas, and Higuchi. The second-order and Korsmeyer-Peppas models were ranked highest, indicating superior performance (R^2^ > 0.96) in describing SP release at intestinal pH compared to other evaluated models. The highest fit to the second-order kinetic model indicates a complex mechanism of the enzyme release process, dependent on both the hydrogel matrix and the enzyme concentration [60]. In the Korsmeyer−Peppas model, the controlled route of drug release is studied using the release exponent n, i.e., Fickian diffusion (*n* ≤ 0.43), non-Fickian release (*n* between 0.43 and 0.85), super case II transport (*n* > 10), and case II transport (n ranged between 0.85 and 1.0) [46]. In our case, the determined n value is less than 0.45 (*n* = 0.30), suggesting that Fickian diffusion process was probably responsible for SP release in the IN-Na^+^ Alg hydrogel. SEM analysis reveals that the hydrogel pore size increases with increasing pH, and the beads disintegrate at a pH value of 7.4, confirming the pH-responsive nature. The TGA study depicts that the material can be stored at normal temperature, and its structural stability cannot be affected by harsh thermal conditions. The hydrogel beads exhibit no antibacterial activity against *B. subtilis* and *E. coli*. The absence of antibacterial activity against *B. subtilis* is due to the prebiotic activity of the inulin, which promotes the growth of this beneficial gram-positive bacterium. These findings further confirmed the prebiotic potential of inulin rather than its antimicrobial effects [27]. Inulin beads also show limited antimicrobial activity against *E. coli*, consistent with their recognized role as a prebiotic rather than targeting specific bacterial strains like *E. coli* [61]. Results of hemolytic activity display the non-toxic nature of the test sample, suggesting that it is safe for use as a drug delivery vehicle.

## 5. Conclusions

In this study, IN-Na^+^ Alg-SP beads were successfully prepared via ionotropic gelation for the controlled release of *Bacillus subtilis* MS1 serine protease. The developed system of hydrogel enables sustained release at intestinal pH and showed strong behavior of pH response while preserving the enzyme under acidic conditions. This highlights its capability to provide a platform for oral delivery of proteins. The developed formulation improved the availability and longevity of the entrapped SP in the intestinal environment. The hydrogel beads are highly sensitive to pH, releasing just a minute quantity of the enzyme at pH 1.2, 3.5, and 4.5. However, at pH 7.4, almost all the enzyme was released due to increased swelling and disintegration of the hydrogel. This distinctive behavior makes the hydrogel effective for shielding drugs in the acidic environment and facilitating their release in the neutral pH of the intestine. The second-order and the Korsmeyer−Peppas model are the best-fitting models (R^2^ > 0.96), describing serine protease release at intestinal pH compared to other evaluated models. The results of the computational docking study further validated the in vitro and ex vivo studies. In conclusion, these hydrogel beads have the potential to be used as an oral drug delivery system for proteins.

Computational docking suggested possible interaction modes with TMV coat protein and a serine protease receptor.

## Figures and Tables

**Table 1:** Different formulations of IN-Na^+^ Alg beads.

Formulations	Inulin (g)	Na^+^ Alginate (g)	Results
1:2	3	6	Fragile bead form
1:1	6	6	Well-formed beads
2:1	12	6	Stiff bead form

**Table 2:** Entrapment efficiency of SP.

Protein Entrapped in an Inulin Bead	Entrapment Efficiency (%)	Apparent Activity of the Enzyme (U)	Specific Activity of the Enzyme (U/mg)	Specific Activity of the Entrapped Enzyme	Relative Activity (%)
3 mg	54	260	3.12	3.22	103

**Table 3:** Kinetic parameters of SP release from the IN-Na^+^ Alg matrix.

Models	Parameters	Drug Release Values
Zero-Order	k	25.4
	R^2^	0.9103
First-Order	k_1_	0.31
	R^2^	0.9577
Second-Order	k_2_	0.0041
	R^2^	0.9697
Higuchi	k_H_	75.604
	R^2^	0.9588
Korsmeye−Peppas	n	0.3063
	k_K-P_	0.6419
	R^2^	0.9692

## Data Availability

The original contributions presented in this study are included in the article. Further inquiries can be directed to the corresponding author.
